# The Modern Treatment of Ringworm

**Published:** 1921-05-21

**Authors:** 


### THE MODERN TREATMENT OF RINGWORM.

When dealing with a condition so common in institutional practice and yet open to so many fallacies in treatment, a book which clearly demonstrates modern methods is always valuable. In the second edition of "Skin Diseases in General Practice,"* Haldin Davis has performed this service particularly well. In that it causes greater loss of educational time among children than any other juvenile disorder, ringworm is a more important disease than seems at first sight probable. The vast majority of cases are due to two species of human fungus, the small-spored (*Microsporon Audouini*) and the large-spored (*Trichophyton endothrix*). Contrary to popular belief, a very small percentage are due to animal forms. In this country the microsporon is predominant, and it is quite exceptional to find a large-spored case in a London child. On the other hand, trichophyton infections form the majority on the Continent. The typical lesions produced are too well known to need description, so we may proceed immediately to consider the treatment. Broadly we may divide it into two sections, namely, with and without *x*-rays.

### X-ray Methods.

There is little doubt that for the great majority of cases seen in elementary schools and hospitals, where as a rule the infection has laid firm hold before its presence is detected, the *x*-rays form by far the best method. Dr. Davis explains that the correct dosage for any part of the scalp is best measured by placing a Sabouraud pastille at a distance from the source of the rays one half as great as the distance of the scalp from the rays. The pastille darkens and when a correct match to a standard tint is obtained the treatment is suspended. In this way epilation, which is necessary, is obtained, and yet permanent baldness should not be caused. The exact measurement of the dose is therefore most important. The author himself uses an accurate instrument known as Corbett's radiometer to be as sure as possible.

A point not always realised is that it is usually best, even if there be only one "patch" visible, to epilate the whole head. Otherwise it frequently occurs that in the interval between exposure to the rays and the falling of the hair, other patches may be infected and ultimately the whole scalp will have to be treated. Only the minimum dose required to make the hair fall out should be used, because, apart from the requirements of safety, the hair then returns much more quickly than when full doses are given. The chief pitfalls in this treatment are, however, (1) *x*-raying the same area twice, (2) giving too large a dose, and (3) giving an insufficient dose. The last is annoying but not harmful, and it is against (1) and (2) that strict adherence to a routine procedure in all cases and correct dose measurement are the only safeguards. It must be remembered too that if an area has once been treated, it must not be again exposed to the rays for at least four weeks. A word of warning is also given against treating an impetiginous case with *x*-rays. This condition is greatly aggravated by them, often with particularly unfortunate results.

### Local Treatment.

Only very early cases will really yield to painting with tincture of iodine. In the commoner class, where the hairs are already involved, Dr. Davis urges remedies which cause active inflammation. Countless remedies have been tried on this principle. Chrysarobin must be very carefully applied, or the effect is far too severe.

The same applies to mercury ointments, and a simpler method is repeatedly to rub in equal parts of common salt and vaseline, bathing the ointment off daily. In this way a gradual inflammation arises, but the process is often painful and the patches must afterwards be treated with dilute ammoniated mercury ointment to allay it . Patches have been dealt with by needling the single follicles with croton oil, but much attention is required and the method is not very practicable. In whatever way the necessary inflammation and removal of the hairs is produced it is far inferior to the application of *x*-rays. But if the rays be used, then it is essential that a highly practised worker must administer them.

* Published by Henry Frowde and Hodder & Stoughton. 25s. net.

